# Mannan endo-1,4-β-mannosidase from *Kitasatospora* sp. isolated in Indonesia and its potential for production of mannooligosaccharides from mannan polymers

**DOI:** 10.1186/s13568-017-0401-6

**Published:** 2017-05-19

**Authors:** Nanik Rahmani, Norimasa Kashiwagi, JaeMin Lee, Satoko Niimi-Nakamura, Hana Matsumoto, Prihardi Kahar, Puspita Lisdiyanti, Yopi, Bambang Prasetya, Chiaki Ogino, Akihiko Kondo

**Affiliations:** 1 Research Center for Biotechnology, Indonesian Institute of Sciences, Komplek CSC-LIPI, Jl. Raya Bogor Km.46, Cibinong, 16911 West Java Indonesia; 2 Graduate School of Science, Technology and Innovation, Kobe University, 1-1 Rokkodaicho, Nada-ku, Kobe, 657-8501 Hyogo Japan; 3 Department of Chemical Science and Engineering, Graduate School of Engineering, Kobe University, 1-1 Rokkodaicho, Nada-ku, Kobe, 657-8501 Hyogo Japan; 4 RIKEN Center for Sustainable Resource Science, 1-7-22 Suehiro-cho, Tsurumi-ku, Yokohama, 230-0045 Kanagawa Japan

**Keywords:** Mannan endo-1,4-β-mannosidase, Mannooligosaccharides (MOS), Screening, *Kitasatospora* sp., *Streptomyces lividans* 1326

## Abstract

Mannan endo-1,4-β-mannosidase (commonly known as β-mannanase) catalyzes a random cleavage of the β-d-1,4-mannopyranosyl linkage in mannan polymers. The enzyme has been utilized in biofuel production from lignocellulose biomass, as well as in production of mannooligosaccharides (MOS) for applications in feed and food industries. We aimed to obtain a β-mannanase, for such mannan polymer utilization, from actinomycetes strains isolated in Indonesia. Strains exhibiting high mannanase activity were screened, and one strain belonging to the genus *Kitasatospora* was selected. We obtained a β-mannanase from this strain, and an amino acid sequence of this *Kitasatospora* β-mannanase showed a 58–71% similarity with the amino acid sequences of *Streptomyces* β-mannanases. The *Kitasatospora* β-mannanase showed a significant level of activity (944 U/mg) against locust bean gum (0.5% w/v) and a potential for oligosaccharide production from various mannan polymers. The β-mannanase might be beneficial particularly in the enzymatic production of MOS for applications of mannan utilization.

**Electronic supplementary material:**

The online version of this article (doi:10.1186/s13568-017-0401-6) contains supplementary material, which is available to authorized users.

## Introduction

Mannan endo-1,4-β-mannosidase [EC 3.2.1.78] (β-mannanase) is used to catalyze a random cleavage of the β-d-1,4-mannopyranosyl linkage (McCleary and Matheson 1986). β-Mannanase is produced by various organisms, such as bacteria, yeasts, and fungi (Dhawan and Kaur 2007). To date, various species of β-mannanase have been cloned and characterized from these organisms (Yamabhai et al. 2016). Most of the microbial β-mannanases belong to glycoside hydrolase (GH) families 5, 26, and 113, based on their amino acid sequences, and on their structural and mechanistic similarities (Kim et al. 2011).

Mannan polymers are widely distributed in plants as part of the hemicellulose fraction in hardwoods, softwoods, seeds of leguminous plants, and beans (Dhawan and Kaur 2007). The use of β-mannanase is one method to the extensive degradation of these mannan polymers for application in biofuel production from lignocellulose biomass (Ishii et al. 2016), as well as in production of mannooligosaccharides (MOS) (Yamabhai et al. 2016). MOS are non-digestible oligosaccharides that have potential applications in dietary fiber and prebiotics (Asano et al. 2003; Jian et al. 2013). Unfortunately, most of the MOS products are derived from sugar polymers present in the cell wall of *Saccharomyces cerevisiae* yeast. Some researchers have studied the prebiotic effect of MOS from plants and showed the potential use to improve human health, such as promoting the growth of intestinal beneficial microflora, decreasing enteric pathogenic bacteria, and reduction of dietary fat absorption (for example, Van Zyl et al. 2010; Jian et al. 2013; Chauhan et al. 2014). The supply of mannan polymers must meet the market demand for MOS.

Application of β-mannanase to produce MOS might be greatly beneficial if a cheap mannan-rich biomass is used as the substrate. Interestingly, Indonesia has abundant sources of biomass that contain high levels of hetero-mannan, such as palm kernel cake, copra cake, porang potato from the bulbs of porang (*Amorphophallus onchophyllus*), and suweg potato from *Amorphophallus campanulatus*. This fact suggests that the production of MOS based on a cheap mannan-rich biomass can be exactly realized. Therefore, the potential of mannan polymers from these biomasses in Indonesia has received an industrial focus, such as the food and feed industry.

β-Mannanase could be naturally produced by actinomycetes. Actinomycetes produce extracellular enzymes that can decompose different types of substrates, and some enzymes from actinomycetes can be employed as important enzyme accessories in industrial processes, such as cellulase, xylanase, pectinase, protease, and chitinase (Prakash et al. 2013). There are several reports related to the cloning and expression of β-mannanase genes from actinomycetes. Fortunately, Indonesia is also rich in terrestrial microbes, particularly in actinomycetes. Several scientists have reported that actinomycetes isolated from Indonesian soils and leaf-litters represent new species and demonstrate genus diversity (for example, Otoguro et al. 2009, 2011; Lisdiyanti et al. 2010; Yamamura et al. 2010). Extensive exploration of industrially useful enzymes has been conducted from these actinomycetes, such as inulin fructotransferase (Pudjiraharti et al. 2011, 2014). Accordingly, there are two impacts using β-mannanases from these actinomycetes for the industrial production of MOS; the first is the possibility to obtain many kinds of β-mannanases with varied enzymatic properties due to the variety of actinomycetes strains, and the second is the high potential production of various MOS from mannan polymers due to the readiness of many kinds of β-mannanases with a broad substrate specificity.

Herein, we report the screening and cloning of a β-mannanase from actinomycetes isolated in Indonesia to explore its possible utilization as mentioned above. We selected one strain (ID04-0555) from the genus *Kitasatospora*, which represents the first trial, as a mannan-degrading enzyme producer. After cloning a β-mannanase gene from this strain, we found that the enzyme demonstrated the release of various types of oligosaccharides from the mannan polymers (particularly substrate derived from raw mannan polymers), indicating its beneficial potential in the production of MOS from raw biomass.

## Materials and methods

### Strains, materials, and chemicals

The actinomycetes were obtained from Biotechnology Culture Collection (BTCC), Indonesian Institute of Sciences (LIPI)*. Streptomyces lividans* 1326 (NBRC 15675) was purchased from National Institute of Technology and Evaluation (NITE, Chiba, Japan). *Escherichia coli* JM109 (Takara, Shiga, Japan) was used as the host strain for DNA manipulation. Locust bean gum (LBG) was purchased from Sigma-Aldrich (St. Louis, MO, USA). Ivory nut and konjac glucomannan were purchased from Megazyme (Wicklow, Ireland). Porang potato was purchased from PT Ambico (Surabaya, East Java, Indonesia). Suweg potato was purchased from a traditional market in East Java, Indonesia. Copra cake and palm kernel cake were purchased from a traditional market in Lampung, Sumatra, Indonesia. Mannose (M1) was purchased from Sigma-Aldrich (St. Louis, MO, USA). Mannobiose (M2), mannotriose (M3), mannotetraose (M4), mannopentaose (M5), and mannohexaose (M6) were purchased from Megazyme (Wicklow, Ireland).

### Screening of actinomycetes strains for a mannan degrading enzyme from actinomycetes

500 strains were cultured on ISP2 agar medium. A single colony was pre-cultured in ISP2 broth at 28 °C, 190 rpm for 3 days, then inoculated into 10 mL modified ISP2 broth which contained 0.4% yeast extract, 1.0% malt extract, and 0.5% each of mannan biomass (LBG, porang potato, copra cake, palm kernel cake, and suweg potato) in 100 mL Erlenmeyer flasks. The fermentation was carried out on a rotary shaker at 28 °C and 190 rpm for 5 days. Sampling was carried out every 24 h. Culture supernatants were collected by centrifugation at 5000 rpm for 10 min. The analysis of β-mannanase activity against mannan substrates was measured on LBG agar (0.5% LBG and 1.8% agar) by individually spotting an aliquot of the culture supernatant (5 µL). The plates were incubated at 37 °C for 3 days. After 3 days, the plates were flooded with an aqueous solution of 0.25% Congo red (Nacalai, Kyoto, Japan) for 30 min to visualize the hydrolysis zones. The plates were then washed twice with 1 M NaCl for 15 min and 0.5% acetic acid to check the LBG degradation more clearly.

The hydrolysis of mannan substrates by the culture supernatant from ID04-0555 strain (LBG as a carbon source) was analyzed via TLC. Hydrolysis of 0.4% (w/v) LBG, 0.2% (w/v) ivory nut, 0.2% (w/v) konjac glucomannan, or 0.3% (w/v) porang was performed containing 50 mM MES buffer (pH 6.5) and 1.0 U/mL of β-mannanase. Reaction mixtures were incubated for 0, 1, 2, 3, and 4 h in a shaker incubator at 30 °C. Reaction mixtures were heated at 100 °C for 5 min to terminate the reaction at various reaction times (0, 1, 2, 3, and 4 h). Reaction mixtures were spotted on TLC Silica gel 60F_254, 20–20 cm_ (EMD/Merck, Darmstadt, Germany) and developed with a mixture of n-Butanol/Acetic Acid/water (2:1.1:1, v/v/v). Spots were stained using DAP that contained diphenylamine, aniline, acetone, and phosphoric acid (Merck KGaA, Darmstadt, Germany), and subsequently heated at 120 °C for 15 min. Mannose (M1), mannobiose (M2), mannotriose (M3), mannotetraose (M4), mannopentaose (M5), and mannohexaose (M6) were used as standards.

### Molecular identification of the ID04-0555 strain

Molecular identification of the ID04-0555 strain was conducted based on the 16S rRNA gene as established by Lisdiyanti et al. (2010). The 16S rRNA gene was amplified via polymerase chain reaction (PCR) technique using a pair of 9F and 1510R primers (Additional file 1: Table S1) (Burggraf et al. 1992). The sequence was confirmed via ABI 3130 DNA sequencer (Applied Biosystems, Foster City, CA, USA) and then compared with others available in the GenBank/DDBJ/EMBL database using multiple sequence alignment (ClustalW).

### Molecular cloning of a β-mannanase gene from the ID04-0555 strain

The ID04-0555 strain was cultured on inorganic salt starch agar plates. A single colony was cultured in TSB medium (17 g/L pancreatic digest of casein, 3.0 g/L enzymatic digest of soya bean contain papain, 2.5 g/L glucose, 5.0 g/L sodium chloride, and 2.5 g/L di-potassium hydrogen phosphate, Oxoid, Hampshire, England) for genomic DNA extraction. Genomic DNA was extracted using the method established by Kieser et al. (2000). A detailed method for cloning the β-mannanase gene appears in Additional file 1.

### Expression and purification of recombinant β-mannanase in *S. lividans* 1326

*Streptomyces lividans* 1326/pUC702-pro-ManKs-(His)_6_ (the recombinant β-mannanase expression strain) was inoculated into a test tube containing 5 mL of TSB medium (Becton, Dickinson and Company, Sparks, MD, USA) supplemented with 5 μg/mL of thiostrepton (EMD chemicals, San Diego, CA, USA), followed by cultivation at 28 °C for 2 days. Then, 1 mL of the seed culture was transferred into a baffled 500 mL shaking flask containing 100 mL of modified TSB medium with 30 g/L glucose (Nacalai, Kyoto, Japan) as a carbon source, 15 g/L tryptone (Nacalai, Kyoto, Japan) as a nitrogen source, and 5 μg/mL of thiostrepton. Cultivation was performed at 28 °C for 3 days.

Recombinant His-tagged β-mannanase was purified using Ni Sepharose™ excel (GE Healthcare, Uppsala, Sweden) according to the manufacturer’s instructions. Homogeneity and molecular mass of the purified β-mannanase were evaluted via 12% SDS polyacrylamide gel electrophoresis. Visualization of the protein bands was accomplished by staining with Coomassie Brilliant Blue G-250 (Nacalai, Kyoto, Japan). For western blotting, proteins were electroblotted onto Immobilon-P transfer membrane (Merck Millipore, Cork, Ireland) from SDS polyacrylamide gel and the His-tagged β-mannanase was allowed to react with Anti-His-tag HRP-DirecT (KDX, Aichi, Japan). The purified β-mannanase protein was electroblotted onto the transfer membrane, stained by Ponceau S (Nacalai, Kyoto, Japan), and analyzed using a peptide sequencer (Procise 492-HT Protein Sequencer, Applied Biosystems). Protein concentration was determined via Quick Start Bradford Protein Assay (Bio-Rad, Hercules, CA, USA) using bovine serum albumin as a standard.

### Enzyme assays

Standard β-mannanase activity was assayed via the 3,5-dinitrosalicylic acid (DNS) method (Miller 1959). The standard reaction was conducted at 45 °C after exactly 15 min in 0.5 mL of a reaction mixture that contained appropriately diluted recombinant enzyme, 0.5% (w/v) LBG, and 50 mM MES buffer (pH 6.5). The amount of reducing sugars liberated in the enzyme reaction was assayed by mixing 0.5 mL of the DNS solution. The mixture was heated at 100 °C for 15 min and cooled on ice. The absorbance of the sample was measured at 540 nm. One unit of β-mannanase activity is defined as the amount of enzyme that liberates 1 µmol of reducing sugar per minute under a given set of experimental conditions. This experiment was repeated three times.

### Characterization of β-mannanase activity

To determine the optimal pH of β-mannanase activity, evaluations were conducted between pH 4.0 and 10 under standard assay conditions using the following buffers: 50 mM Acetate buffer (pH 4.0–5.0), MES buffer (pH 5.0–7.0), MOPS buffer (pH 7.0–8.5), and glycine–NaOH buffer (pH 8.5–10), respectively. The optimal temperature of activity was evaluated by incubating the enzyme samples with the substrate at temperatures ranging from 30 to 90 °C in 50 mM MES buffer (pH 6.5). Thermal stability of the activity was determined by incubating the enzyme in 50 mM MES buffer (pH 6.5) at 45, 50, and 55 °C for a maximum of 120 min. The effect of various metal ions and chemical reagent on the activity was determined by incubating the enzyme in 50 mM MES (pH 6.5) containing 0.5% (w/v) LBG in the presence of 1 mM of CaCl_2_, CuCl_2_, CoCl_2_, NaCl, KCl, MgCl_2_, MnCl_2_, and ZnCl_2_, and 5 mM of EDTA. These experiments were repeated three times.

Relative activity of β-mannanase against LBG, ivory nut, konjac glucomannan, and porang potato was determined by 0.5% (w/v) of each substrate in 50 mM MES buffer (pH 6.5) at 45 °C for 15 min. For determination of the kinetic parameters, concentrations of LBG were varied from 0.05 to 0.5% (w/v) and the activity of the recombinant β-mannanase was monitored in 50 mM MES buffer (pH 6.5) at 45 °C for 30 min. Michaelis–Menten parameters (*V*_max_ and *K*_m_ values) were estimated by the Hanes–Woolf plot. These experiments were repeated three times.

The hydrolysis products from mannan polymers by the recombinant β-mannanase were analyzed via TLC. Details of this method were shown earlier in materials and methods. Hydrolysis of mannan polymers was performed containing 50 mM MES buffer (pH 6.5) and 1.0 U/mL of the recombinant β-mannanase. Reaction mixtures were incubated for 0, 1, 2, 3, 4, 24, 48, and 72 h at 30 °C. Additionally, hydrolysis of 0.5% (w/v) M1–M6 was performed containing 50 mM MES buffer (pH 6.5) and 3.1 U/mL of the recombinant β-mannanase.

### Nucleotide sequence accession number

The nucleotide sequence of the β-mannanase gene isolated from the ID04-0555 strain (BTCC B-806, GenBank database under the accession number KY576672) has been deposited in the GenBank database under the accession number LC012037.

## Results

### Screening using mannan polymers

**Fig. 1:**
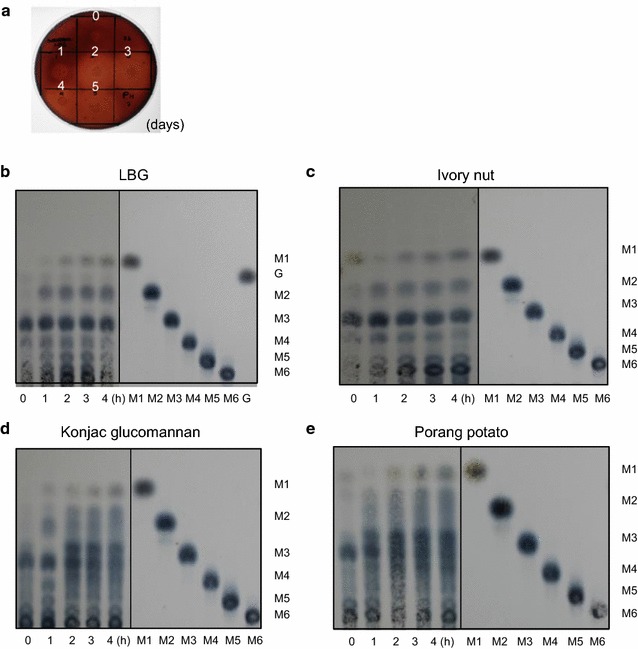
**a** Clear zone on LBG agar medium at pH 7.0 using culture supernatant of ID04-0555 strain, indicating hydrolysis of β-mannanase (0, 1, 2, 3, 4, and 5 day cultivation). **b**–**e** TLC analysis of hydrolysis products from various mannan substrates using secreted mannan degradation enzymes of ID04-0555, **b** LBG, **c** ivory nut, **d** konjac glucomannan, and **e** porang potato. Standards (STD), mannose (M1), mannobiose (M2), mannotriose (M3), mannotetraose (M4), mannopentaose (M5), mannohexaose (M6), and galactose (G)

At first, we screened 500 isolates to identify strains exhibiting high mannanase activity among the actinomycetes collected by biotechnology culture collection (BTCC) from Indonesian soils and leaf-litters. These strains were cultivated in the ISP2 medium using LBG as a carbon source, and qualitative analysis of hydrolysis activity was performed using Congo red dye (such as ID04-0555 strain shown in Fig. 1a). One strain, the ID04-0555 strain, was selected with the high activity among these isolates. We also confirmed that the hydrolysis activity of the ID04-0555 strain was induced by various sources of Indonesian mannan biomass, such as porang potato, copra cake, palm kernel cake, and suweg potato substrates. (Additional file 1: Figure S1).

To investigate hydrolytic patterns of the secreted mannan degrading enzymes from the ID04-0555 strain, reactions were measured based on TLC analysis using the culture supernatant. The culture supernatant from the ID04-0555 strain was able to hydrolyze mannan polymers into oligosaccharides (Fig. 1b–e). LBG was hydrolyzed into mannose and various types of oligosaccharides for 4 h, and trisaccharide was one of the major products. A similar degradation pattern was observed using ivory nut. The hydrolysis of glucomannan, such as the konjac glucomannan and porang potato, also produced various oligosaccharides with trisaccharides as main products.

### Molecular identification of the ID04-0555 strain

**Fig. 2:**
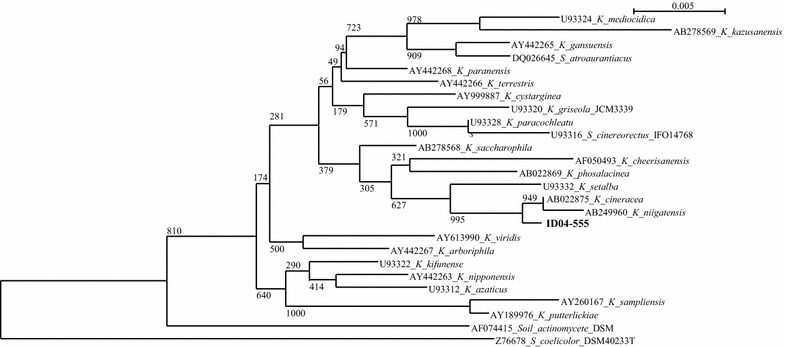
Neighbor-joining phylogenetic tree of genus *Kitasatospora* and positions of ID04-0555 strain (KY576672_*K*_BTCC B-806) based on 16S rRNA gene sequence analysis. Bootstrap values (>50 %) based on 1000 replicates are shown at the branch nodes. The sequence of *Streptomyces coelicolor* DSM 40233^T^ was used as the outgroup. *Bar* 0.005 substitutions per nucleotide position. Representative sequences in the dendrogram were obtained from GenBank (accession number in parentheses)

The ID04-0555 strain was isolated in a soil sample collected from Cibinong, West Java, Indonesia. Phylogenetic analysis confirmed that this strain belongs to the family *Streptomycetaceae* and to the genus *Kitasatospora*. Moreover, neighbor-joining phylogenetic analysis based on a 16S rRNA gene sequence revealed that the strain belongs to the genus *Kitasatospora* and is closely related to *Kitasatospora cineracea* and *Kitasatospora niigatensis* (97% sequence similarity) (Fig. 2).

### Cloning and sequence analysis of a β-mannanase gene from the ID04-0555 strain

**Fig. 3:**
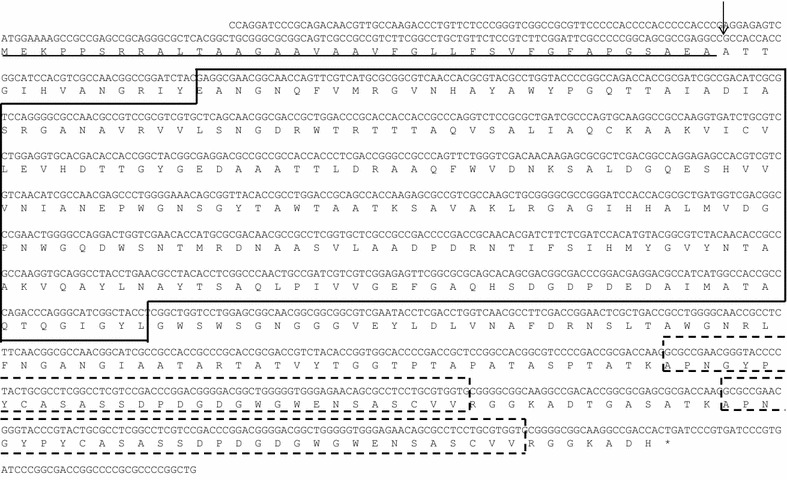
Nucleotide and deduced amino acid sequences of ManKs_4-555 from ID04-0555. Stop codon is indicated by the *asterisk*. The *underline* and *down arrow* represent the signal peptide and cleavage site, respectively. The *box* and the *dotted boxes* represent putative catalytic domain and putative carbohydrate-binding modules, respectively

We amplified a partial β-mannanase gene from the ID04-0555 strain via a PCR reaction with a set of primers designed by β-mannanase sequences of five *Streptomyces* strains from NCBI/NBRC. Then, an entire open reading frame of the β-mannanase sequence was identified from the partial gene sequence (for details of the method, see Additional file 1). We obtained 1435 nucleotides in the DNA sequence, which revealed an open reading frame (ORF) of 1302 nucleotides encoding a protein with 434 amino acids and a calculated molecular mass of 41.4 kDa (referred to as ManKs_4-555) (Fig. 3).

Alignment of the putative amino acid sequence showed that ManKs_4-555 was comprised of a putative N-terminal catalytic domain (from E51 to L288) and two cellulose binding modules (CBMs) at the C-terminus region (from A355 to V384 and from A398 to V427). The putative catalytic domain of ManKs_4-555 resembles the domain structure of a glycoside hydrolase family 5 (GH5). The carbohydrate-binding modules are similar to those of family 10 CBMs (Fig. 3).

### Secreted expression of ManKs_4-555 in a heterologous host

The ManKs_4-555 β-mannanase gene, fused to a sequence encoding a histidine tag at the C-terminal, was ligated with the pUC702 vector under the control of the *Streptoverticillium cinnamoneum* phospholipase D promoter (Ogino et al. 2004). The constructed vector (referred to as pUC702-pro-ManKs-(His)_6_, Additional file 1: Table S1) was transformed into *Streptomyces lividans* 1326, which has been employed as a host strain for recombinant protein expression. The transformant strain was named *S. lividans* 1326/pUC702-pro-ManKs-(His)_6_ (Additional file 1: Table S1).

**Fig. 4:**
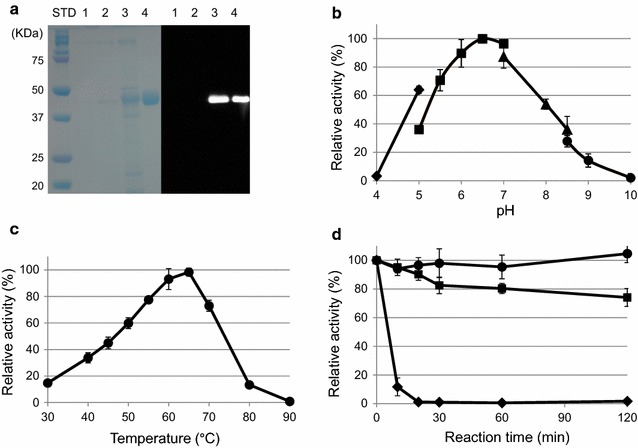
**a** SDS-PAGE and western blotting of recombinant β-mannanase (ManKs_4-555) expressed in *S. lividans* 1326; STD, molecular weight marker; *lane 1*, culture supernatant from *S. lividans* 1326 (wild type); *lane 2*, culture supernatant from *S. lividans* 1326/pUC702-pro; *lane 3*, culture supernatant from *S. lividans* 1326/pUC702-pro-ManKs-(His)_6_ before purification; *lane 4*, purified β-mannanase (ManKs_4-555). **b** Effect of pH on β-mannanase activity of ManKs_4-555 containing 0.5% (w/v) LBG. Activity was measured at 45 °C using the following buffers: acetate buffer (pH 4.0–5.0, *filled diamond*), MES buffer (pH 5.0–7.0, *filled square*), MOPS buffer (pH 7.0–8.5, *filled triangle*), and glycine–NaOH buffer (pH 8.5–10, *filled circle*). ManKs_4-555 activity at pH 6.5 was taken to be 100%. **c** Temperature dependence of ManKs_4-555. Activity was measured at 30–90 °C using 50 mM MES buffer (pH 6.5) containing 0.5% (w/v) LBG. β-Mannanase activity at 65 °C was taken to be 100%. **d** Thermal stability of ManKs_4-555. Activity was measured at 45 °C (*circle*), 50 °C (*square*), and 55 °C (*diamond*) using 50 mM MES buffer (pH 6.5) for a maximum of 120 min. β-Mannanase activity at the starting point was taken to be 100%

After the secreted recombinant β-mannanase was expressed in the host strain, the culture supernatant was analyzed by SDS-PAGE and western blotting (Fig. 4a). A single protein band corresponding to the β-mannanase was observed, whereas no protein band was observed in the supernatant from either *S. lividans* 1326 wild-type strain or *S. lividans* 1326/pUC702-pro strain, which is a transformant harboring the pUC702-pro vector as a control (Fig. 4a; Additional file 1: Table S1). Maximum β-mannanase activities of culture supernatants from *S. lividans* 1326/pUC702-pro-ManKs-(His)_6_, *S. lividans* 1326 wild-type, and *S. lividans* 1326/pUC702-pro strains reached 84, 0.39, and 0.41 U/mL, respectively, after cultivation at 28 °C for 72 h. These results indicate that recombinant β-mannanase was expressed in the *S. lividans* 1326/pUC702-pro-ManKs-(His)_6_ strain. The maximum yield was 1.4 mg based on a 100 mL culture scale after purification. N-terminal amino acid sequence analysis (A-T-T-G-I-H-V-A-N-G) using a peptide sequencer indicates that the recombinant β-mannanase had a signal peptide from M1 to A37, and the sequence between A37 and A38 was cleaved during the secretory process.

### Characterization of recombinant ManKs_4-555

The effect of pH on ManKs_4-555 activity was evaluated in 50 mM of various buffers at 45 °C for 15 min using LBG as a substrate. Maximum activity was observed at pH 6.5, and more than 80% maximum activity was detectable in the neutral pH region of 6.0–7.0 (Fig. 4b). Maximum activity was observed at a temperature of 65 °C (pH 6.5), and approximately 100% maximum activity was detectable at 45 °C for a maximum of 2 h (Fig. 4c, d). Slight increases (approximately 1.3- and 1.2-fold of its original activity) in ManKs_4-555 activity were observed in the presence of divalent cations (each 1 mM) such as Co^2+^ and Mn^2+^. ManKs_4-555 activity was inhibited by metal ions such as Cu^2+^ (6.5%), Zn^2+^ (57%), and metal chelator EDTA (38%) (Table 1).

**Table 1:** Effect of various metal ions and chemical reagent on the activity of ManKs_4-555

Reagent	Relative activity (%)
None	100
CaCl_2_	109 ± 2
CuCl_2_	6.5 ± 2.6
CoCl_2_	126 ± 8
NaCl	107 ± 6
KCl	104 ± 2
MgCl_2_	103 ± 7
MnCl_2_	115 ± 12
ZnCl_2_	57 ± 1
EDTA	38 ± 2

Specific activity and kinetic parameters of ManKs_4-555 were determined using LBG as a substrate. The specific activity of the ManKs_4-555 was 944 ± 54 U/mg under standard conditions (as mentioned in the “Materials and methods”). *K*_*m*_ and *V*_*max*_ values of 0.55 mg/mL and 1054 μmol/min/mg were determined using 0.05–0.5% LBG. The hydrolytic activity of the ManKs_4-555 was assayed against various mannan polymers as substrates. The ManKs_4-555 hydrolyzes LBG galactomannan, ivory nut, konjac glucomannan and porang potato glucomannan with a relative hydrolysis of 100, 40, 90, and 65, respectively (Table 2).

**Table 2:** Effect of various substrates on the activity of ManKs_4-555 compared to LBG

Substrate	Relative activity (%)
Locust bean gum	100
Ivory nut	40 ± 3
Konjac Glucomannnan	90 ± 5
Porang potato	65 ± 2

### Hydrolysis of mannan polymers using recombinant ManKs_4-555

**Fig. 5:**
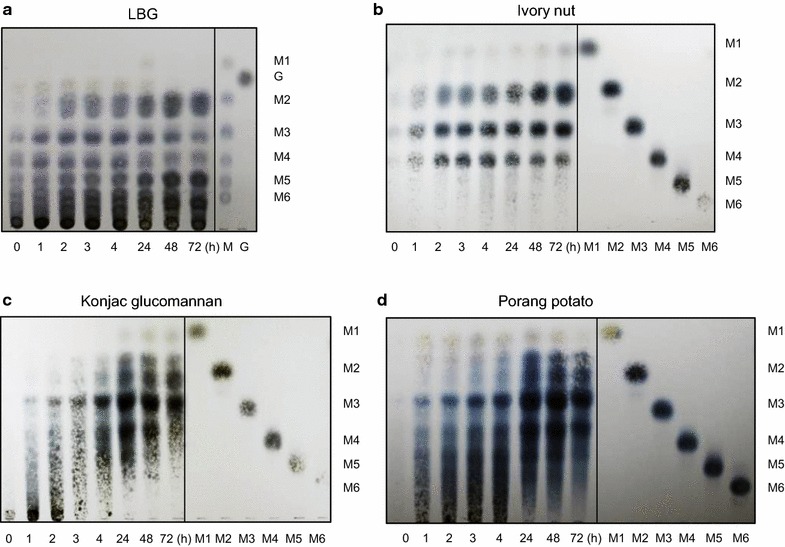
TLC analysis of ManKs_4-555 hydrolysis products from various mannan substrates, **a** LBG, **b** ivory nut, **c** konjac glucomannanan, and **d** porang potato. Standards (STD), mannose (M1), mannobiose (M2), mannotriose (M3), mannotetraose (M4), mannopentaose (M5), mannohexaose (M6), and galactose (G)

To investigate the hydrolytic pattern of mannan polymers, reactions with various mannan substrates were evaluated based on TLC method using recombinant ManKs_4-555, and results are shown in Fig. 5. ManKs_4-555 has the ability to hydrolyze some substrates such as LBG, ivory nut, konjac glucomannan, and porang potato for up to 72 h of reaction time. This β-mannanase produced various length oligosaccharides from LBG (Fig. 5a). On the other hand, it produced mannobiose, mannotriose, and mannotetraose from ivory nut as the main products (Fig. 5b). From glucomannan such as konjac glucomannan and porang potato, the β-mannanase produced oligosaccharides presumably derived from disaccharide, trisaccharide, and tetrasaccharide (Fig. 5c, d). We also analyzed the hydrolysis of mannose and oligosaccharides (from mannobiose to mannohexaose) using the ManKs_4-555 (Additional file 1: Figure S2). The substrates from mannose to mannotetraose were hardly hydrolyzed by the β-mannanase, whereas mannopentaose and mannohexaose were hydrolyzed into mannobiose, mannotriose, and mannotetraose (Additional file 1: Figure S2).

## Discussion

Recently, the mannan degrading enzymes have gained interest for application in feed and food industries by the production of potentially health-promoting mannooligosaccharides (MOS). Here, we cloned and characterized a β-mannanase from actinomycetes to have potential applications for mannan utilization, such as the production of MOS.

In this study, one β-mannanase (referred to as ManKs_4-555, Fig. 3) was cloned from a *Kitasatospora* strain exhibiting high mannanase activity in the production of oligomannan (Fig. 2). A BLAST search indicates that the amino acid sequence of ManKs_4-555 is closely related to that of *Streptomyces* β-mannanases (58–71%), such as *Streptomyces* sp. s6-204 (70%), *S. lividans* 66 (69%) (Arcand et al. 1993), *Streptomyces* sp. S27 (66%) (Shi et al. 2011), *S. thermolilacinus* (58%) (Kumagai et al. 2011), and *Cellulosimicrobium* sp. HY-13 (60%) (Kim et al. 2011) (Additional file 1: Figure S3). Eight catalytic residues are conserved in ManKs_4-555 (Additional file 1: Figure S3).

Characterization studies of ManKs_4-555 revealed that optimal values for pH and temperature were similar to those of *Streptomyces* β-mannanases reported previously (Table 3). On the other hand, the specific activity of ManKs_4-555 using LBG at 45 °C was high compared to other *Streptomyces* β-mannanase, although the enzyme assay was different in each experiment [*S. lividans* 66 (876 U/mg) (Arcand et al. 1993), *Streptomyces* sp. S27 (2107 U/mg) (Shi et al. 2011), *S. thermolilacinus* (61 U/mg) (Kumagai et al. 2011), and *S. ipomoea* CECT 3341 (56 U/mg) (Montiel et al. 2002)] (Table 3). ManKs_4-555 also efficiently hydrolyzed ivory nut and konjac glucomannan, including mannan biomass in Indonesia such as porang potato glucomannan, in addition to LBG (Table 2).

**Table 3:** Homology analysis and enzyme properties of ManKs_4-555 compared with other *Streptomyces* β-mannanases

Microbial source	Enzyme	Homology (%)	Mol. Wt (kDa)	Optimal pH	Optimal temp. (°C)	Specific activity (U/mg)	Reference
ID04-0555	ManKs_4-555^a^		41.4	6.5	65	944	This study
*Streptomyces lividans* 66	β-Mannanase^b^	69	36	6.8	58	876	Arcand et al. (1993)
*Streptomyces* sp. S27	Man5S27^a^	66	37.2	7.0	65	2107	Shi et al. (2011)
*Streptomyces thermolilacinus*	StMan^a^	58	–	6.0–8.0	55	61	Kumagai et al. (2011)
*Streptomyces ipomoea* CECT 3341	Man3^b^	–	40	7.5	55	56	Montiel et al. (2002)

^a^Recombinant enzyme

^b^Purified enzyme

The activity of ManKs_4-555 was enhanced with Co^2+^ and Mn^2+^ ions (Table 1). The activity of some *Streptomyces* β-mannanases was reported to be enhanced with Mn^2+^ ion (Shi et al. 2011; Montiel et al. 2002), whereas the activity of these β-mannanases was not reported to be enhanced with Co^2+^ ion. *Cellulosimicrobium* sp. strain HY-13 β-mannanase, which has amino acid sequences corresponding to ManKs_4-555, showed a similar tendency toward enhanced activity with Co^2+^ and Mn^2+^ ions (Kim et al. 2011). In contrast, the activity of ManKs_4-555 was inhibited with Zn^2+^ ion (Table 1). Several β-mannanases from *Streptomyces* strains (Kumagai et al. 2013) and *Cellulosimicrobium* sp. (Kim et al. 2011) had a similar tendency to inhibit activity with Zn^2+^ ion. The ManKs_4-555 is presumably sensitive to metal ion, because the activity of the β-mannanase was inhibited with EDTA (Table 1).

Recombinant ManKs_4-555 produced oligosaccharides from mannan polymers, including various length oligosaccharides from LBG (Fig. 5a) and mannobiose, mannotriose, and mannotetraose mainly from ivory nut (Fig. 5b). ManKs_4-555 was different from the extracellular enzymes of the wild-type ID04-0555 strain in the production of oligosaccharides (trisaccharides as the main product from the enzymes of the ID04-0555 strain in Fig. 1b, c). Other mannan degradation enzymes might be also secreted from the ID04-0555 strain with the exception of the ManKs_4-555. In addition, the mannan degradation pattern for ManKs_4-555 was different from other previously reported *Streptomyces* and *Cellulosimicrobium* β-mannanases showing amino acid sequences corresponding to ManKs_4-555. For example, *Streptomyces* sp. S27 β-mannanase produced trisaccharide (22.14%) as the main product from LBG along with mannose to pentasaccharide (Shi et al. 2011), and *S. thermolilacinus* β-mannanase produced disaccharide and trisaccharide from LBG (Kumagai et al. 2011). The *Cellulosimicrobium* sp. strain HY-13 β-mannanase produced mannan polysaccharide to primarily pentasaccharide from LBG, but the β-1,4-mannanase produced M_4_ (25.2%) and various length oligosaccharides from ivory nut mannan (Kim et al. 2011). Some β-mannanases other than actinomycetes were reported to produce mainly mannobiose and mannotriose from ivory nut mannan (Ademark et al. 1998; Sachslehner and Haltrich 1999; Stålbrand et al. 1993; Jiang et al. 2006). Several reported β-mannanases, such as *Bacillus subtilis* WY34 and *Penicillium occitanis* Pol6, were similar to ManKs_4-555 in the degradation pattern of copra mannan or ivory nut mannan (Jiang et al. 2006; Blibech et al. 2010). ManKs_4-555 showed the production of oligosaccharides from various mannan substrates, and this feature might be useful for the production of MOS based on mannan biomass.

In conclusions, a *Kitasatospora* strain exhibiting high mannanase activity in the production of oligomannan was screened from actinomycetes strains isolated in Indonesia. A β-mannanase was then cloned from the strain and characterized. The amino acid sequence of the *Kitasatospora* β-mannanase showed a 58–71% similarity with the amino acid sequences of *Streptomyces* β-mannanases. Several characteristics, such as high hydrolysis activity and production of oligosaccharides from various mannan including raw mannan polymers, were found to be specific to the *Kitasatospora* β-mannanase, and these are thought to have important practical applications. Such properties suggest the potential utilization of ManKs_4-555 (or also the mother strain) for research in biodegradation of raw mannan biomass and prebiotics. We examined an actinomycete strain not yet previously reported for the cloning of β-mannanase. The investigation of different actinomycetes strains might be potentially useful for obtaining β-mannanases with varied enzymatic properties.

## Additional file

**Additional file 1.** Additional Figures and Tables.

## Abbreviations

MOS: mannooligosaccharides
GH: glycoside hydrolase
CBM: carbohydrate-binding module
DNS: 3,5-dinitrosalicylic acid
LBG: locust bean gum
