# Supplementary material for: Mannan endo-1,4-β-mannosidase from Kitasatospora sp. isolated in Indonesia and its potential for production of mannooligosaccharides from mannan polymers
Source: AMB Express. 2017 May 19;7:100. doi: 10.1186/s13568-017-0401-6 (PMC5438323; doi:10.1186/s13568-017-0401-6)
Supplement: Supplementary file 1 — Additional file 1. Additional Figures and Tables. [file 13568_2017_401_MOESM1_ESM.docx]

Mannan endo-1,4-β-mannosidase from *Kitasatospora* sp. isolated in Indonesia and its potential for production of mannnooligosaccharides from mannan polymers

Nanik Rahmani^1^, Norimasa Kashiwagi^2^, JaeMin Lee^3^, Satoko Niimi-Nakamura^3^, HanaMatsumoto^3^, Prihardi Kahar^3^, Puspita Lisdiyanti^1^,Yopi^1^, Bambang Prasetya^1^,

Chiaki Ogino^3,*^, Akihiko Kondo^2,4^

^1^ Research Center for Biotechnology, Indonesian Institute of Sciences, Komplek CSC-LIPI, Jl. Raya Bogor Km.46, Cibinong 16911, West Java, Indonesia

^2^ Graduate School of Science, Technology and Innovation, Kobe University, 1-1 Rokkodaicho, Nada-ku, Kobe, Hyogo 657-8501, Japan

^3^ Department of Chemical Science and Engineering, Graduate School of Engineering, Kobe University, 1-1 Rokkodaicho, Nada-ku, Kobe, Hyogo 657-8501, Japan

^4^ RIKEN Center for Sustainable Resource Science, 1-7-22 Suehiro-cho, Tsurumi-ku, Yokohama, Kanagawa 230-0045, Japan

* Corresponding author:

*E-mail*: [ochiaki@port.kobe-u.ac.jp](mailto:ochiaki@port.kobe-u.ac.jp)

**Figures**


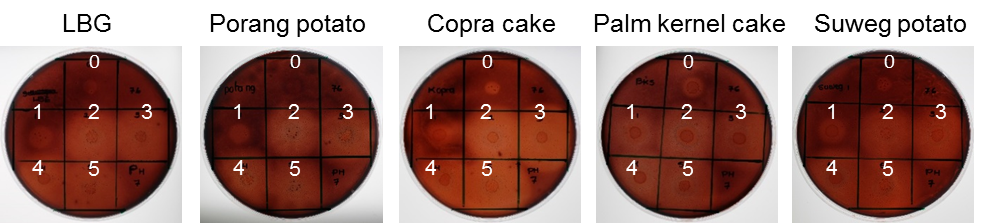


**Figure S1** Clear zone on the LBG agar medium at pH 7.0 using culture supernatant of the ID04-0555 strain (0, 1, 2, 3, 4, and 5 d cultivation) using various types of mannan biomass (LBG, porang potato, copra cake, palm kernel cake, and suweg potato) as carbon sources indicating the hydrolysis of β-mannanase.


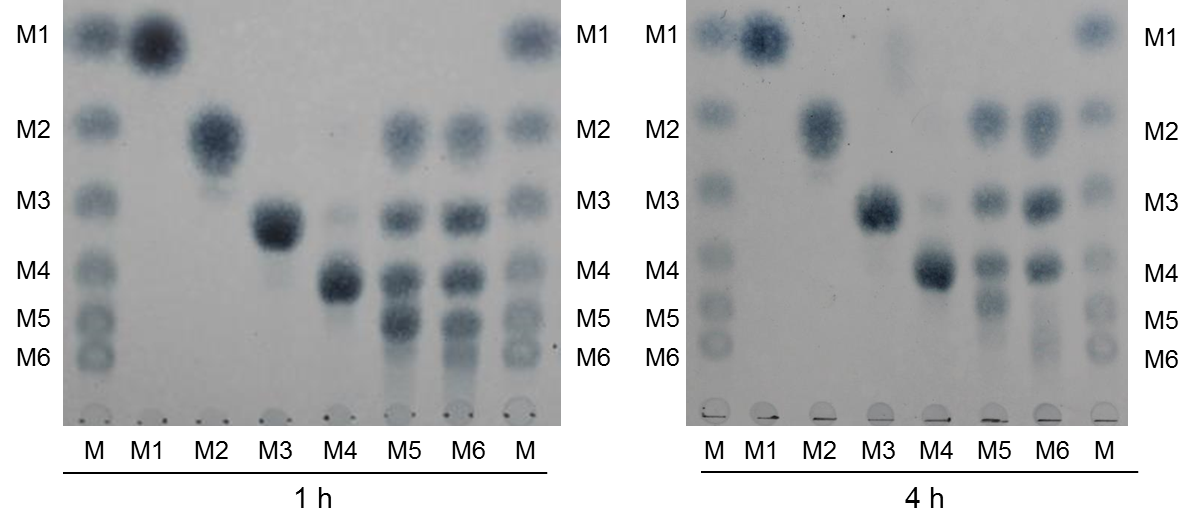


**Figure S2** TLC analysis of hydrolysis products from mannose and oligomannans (from mannobiose to mannohexaose) using recombinant ManKs_4-555 for 1 and 4 h. Each substrate concentration was 0.5% in 50 mM MES buffer (pH 6.5) at 30^o^C.


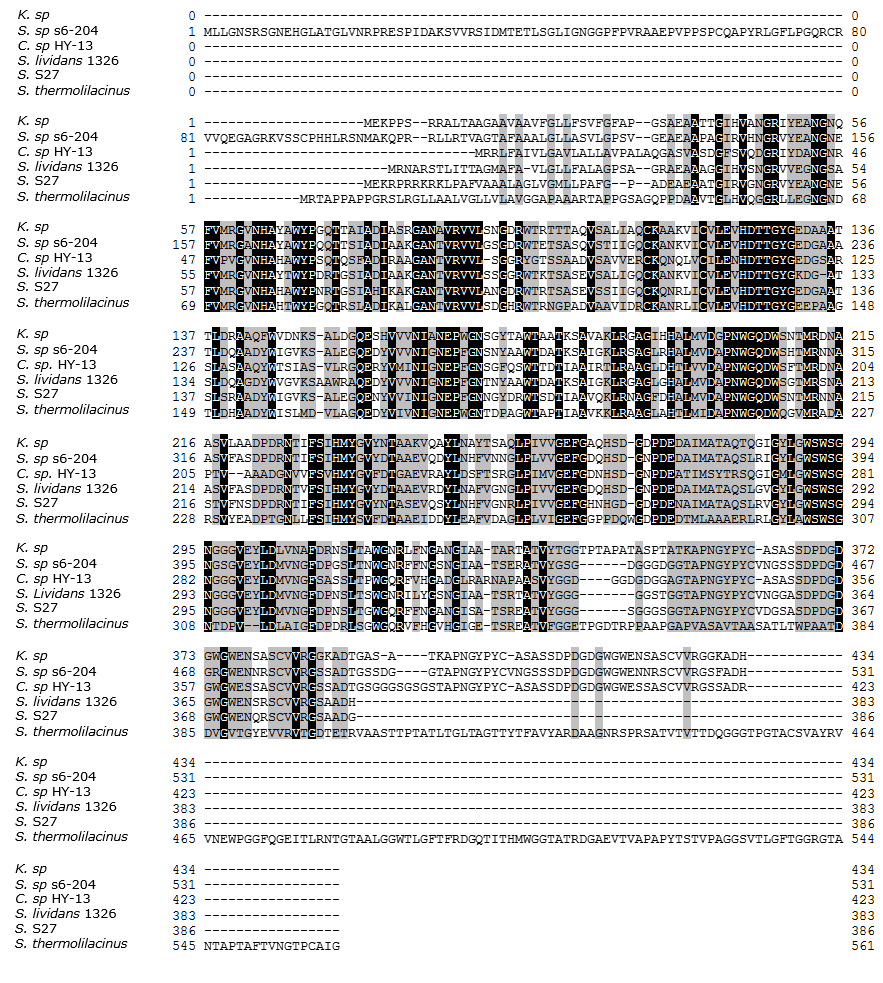


*

*

*

*

*

*

*

*

**Figure S3** Multiple alignment of amino acid sequences for the β-mannanase from *Kitasatospora* sp. Numbers on the left are the residue numbers of the first amino acid in each line. Sequences listed include those of β-mannanase from *Kitasatospora* sp. (*K. sp*) in this study, *Streptomyces* sp. s6-204 (ABY90130), *Cellulosimicrobium* sp. HY-13 (AEE43708), *Streptomyces lividans* (*S. lividans* 1326, AAA26710), *Streptomyces* sp. S27 (ADK91085), and *Streptomyces thermolilacinus* (BAK26781). Identical and similar amino acids are shaded in black and grey, respectively. Highly conserved amino acid residues that play an important role in the catalytic reaction are indicated by asterisk.

**Table**

**Table S1** Plasmids, strains or oligonucleotide primers used in this study.

| Plasmids, strains or oligonucleotide primers | Relevant features |
| --- | --- |
| Plasmids |  |
| pUC702-pro | Versatile vector for protein expression; thiostrepton resistance marker; *pld* promoter; *pld* terminator; *rep*, replication gene from pIJ101; MCS *sph*I-*Nhe*I-*Pst*I-*Hpa*I-*Bgl*II; sizes 8600 bp |
| pUC702-pro-ManKs-(His)_6_ | Vector for secreting β-mannanase; thiostrepton resistance marker; *pld* promoter; *pld* terminator; *rep*, replication gene from pIJ101; sizes 10000 bp |
| Strains |  |
| *Streptomyces lividans* 1326 | WT strain (NBRC 15675) |
| *S.lividans* 1326/pUC702-pro | Transformant harboring pUC702-pro |
| *S.lividans* 1326/pUC702-pro-  ManKs-(His)_6_ | Transformant harboring pUC702-pro-ManKs-(His)_6_ |
| Oligonucleotide primers |  |
| man5A_Ks_univF | GTGCACGACACCACCGGCTAC |
| man5A_Ks_univR | GGACCAGTCCTGGCCCCAGTT |
| S1_up | CGCGCTCTTGTTGTCGACCCAGAACTG |
| S2_up | GTAACCGCTGTTTCCCCAGGGCTCG |
| S1-1_down | CCAGTTCTGGGTCGACAACAAGAGCG |
| S2-1_down | CGAGCCCTGGGGAAACAGCGGTTACAC |
| S1-2_down | TTCGGCGCGCAGCACAGCGACGGCGA |
| S2-2_down | CCCGCACGAGGACGCCATCATGGCCAC |
| man_SphI_F | TAAGGATGCAGCATGGAAAAGCCGCCGAGCCG |
| man_BglII_R_His6 | AGTCGTCTCAAGATCTTCAGTGGTGGTGGTGGTGGTGGTCGGCCTTGCCGCCCC |
| 9F | AGRGTTTGATCMTGGCTCAG |
| 1510R | TACGGYTACCTTGTTAYGACTT |

**Molecular cloning of β-mannanase gene from ID04-0555 strain**

Oligonucleotide primers (man5A_Ks_univF and man5A_Ks_univR) (Table S1) for cloning were designed based on the 250 bp GH5 conserved catalytic region from five types of *Streptomyces* β-mannanase sequences: *S. fradiae* (NCBI accession number HM062520.1), *S. lividans* (NCBI accession number M92297.2), *Streptomyces* sp. s6-204 (NCBI accession number EU399236.1), *S. thermolilacinus* NBRC 14274 (NCBI accession number AB602047.1), and *S. thermoluteus* NBRC 14269 (NCBI accession number AB712236.1). The 250 bp partial fragment of the gene encoding β-mannanase from ID04-0555 chromosomal DNA was amplified by PCR reactions using PrimeSTAR GXL DNA polymerase (Takara, Shiga, Japan) with a pair of the designed oligonucleotide primers. The partial fragment was cloned into PCR^TM^-Blunt II-TOPO® vector (Invitrogen, Carlsbad, CA, USA) and transformed into *E.coli* JM109 cells for sequencing. After confirming the sequence of the partial fragment, the full length of the β-mannanase gene was identified by PCR reactions from the region of the partial fragment. The full length of the gene was identified using both LA PCR^TM^ *in vitro* cloning kit (Takara, Shiga, Japan) and DNA walking SpeedUp^TM^ premix kit (Seegene, Seoul, Korea) according to the manufacturer’s instructions with the following oligonucleotide primers (S1_up, S2_up, S1-1_down, S2-1_down, S1-2_down, and S2-2_down, Table S1). The sequence was confirmed using an ABI3130 DNA sequencer. Finally, the sequence of PCR fragments was assembled to obtain the full length of β-mannanase gene. The full length of β-mannanase gene was amplified by PCR reactions using PrimeSTAR GXL DNA polymerase from the genomic DNA of ID04-0555 by using a pair of primers (man_SphI_F and man_BglII_R_His6) and introduced into the *Sph*I and *Bgl*II sites of pUC702-pro-term (Table S1) using In-Fusion HD Cloning Kit (Takara, Shiga, Japan). The sequence was confirmed using ABI3130 DNA sequencer. The transformation with the recombinant plasmid (pUC702-pro-ManKs-(His)_6_) to *S.lividans* 1326 was conducted according to the method established by Kieser et al. (2000).
